# The role of GABA in type 1 diabetes

**DOI:** 10.3389/fendo.2024.1453396

**Published:** 2024-11-15

**Authors:** Gail J. Mick, Kenneth L. McCormick

**Affiliations:** Department of Pediatrics, University of Alabama at Birmingham, Birmingham, AL, United States

**Keywords:** gamma aminobutyric acid (GABA), Type 1 diabetes, GABA treatment/diabetes, β-cells/pancreatic islets, α-cells/glucagon, diabetes/new therapies, GABA-producing microbes, microbiome/GABA/glutamate

## Abstract

Gamma aminobutyric acid (GABA) is synthesized from glutamate by glutamic decarboxylase (GAD). The entero-pancreatic biology of GABA, which is produced by pancreatic islets, GAD-expressing microbiota, enteric immune cells, or ingested through diet, supports an essential physiologic role of GABA in the health and disease. Outside the central nervous system (CNS), GABA is uniquely concentrated in pancreatic β-cells. They express GAD65, which is a type 1 diabetes (T1D) autoantigen. Glutamate constitutes 10% of the amino acids in dietary protein and is preeminently concentrated in human milk. GABA is enriched in many foods, such as tomato and fermented cheese, and is an over-the-counter supplement. Selected microbiota in the midgut have the enzymatic capacity to produce GABA. Intestinal microbiota interact with gut-associated lymphoid tissue to maintain host defenses and immune tolerance, which are implicated in autoimmune disease. Although GABA is a widely known inhibitory neurotransmitter, oral GABA does not cross the blood brain barrier. Three diabetes-related therapeutic actions are ascribed to GABA, namely, increasing pancreatic β-cell content, attenuating excess glucagon and tamping down T-cell immune destruction. These salutary actions have been observed in numerous rodent diabetes models that usually employed high or near-continuous GABA doses. Clinical studies, to date, have identified positive effects of oral GABA on peripheral blood mononuclear cell cytokine release and plasma glucagon. Going forward, it is reassuring that oral GABA therapy has been well-tolerated and devoid of serious adverse effects.

## 1 Introduction

The pathogenesis of autoimmune type 1 diabetes mellitus (T1D) involves infiltration of the pancreatic islet cells by T-lymphocytes, macrophages, and other immune cells with consequent loss of insulin producing β-cells (1–3). Both genetic susceptibility related to HLA and non-HLA genes as well as environmental factors (infectious, dietary, the microbiome) participate in this process (4, 5). Clinical staging of at-risk subjects according to autoantibodies and dysglycemia has guided potential preventive and therapeutic interventions. At the onset of T1D, more than 70% of β-cells are eradicated (6), thus, residual β-cell replication, intra islet cell transformations and progenitor ductal neogenesis may represent pathways for restoration of β−cell mass. (7). Studies from organ donor pancreata demonstrate insulin-containing islets despite decades following T1D onset (8) indicating ongoing β-cell renewal despite lasting autoimmunity and other stressors. A myriad of immunological abnormalities have been reported in those with T1D including, but not limited to, the production of autoantibodies and cytokines as well as the inability of regulatory T cells (Treg) to curtail the action of effector T cells (Teff); the latter distinct cell population participate in the immune destructive processes. Therefore, a vast majority of clinical studies attempting to curtail this immune foray have focused on immune suppression (9, 10). Additionally, dysfunction in the exocrine pancreas, aberrant sympathetic innervation, oxidative stress, ER stress, and altered autocrine and paracrine signaling within the islet cell are potential therapeutic targets in T1D (11–17).

**Figure 1:**
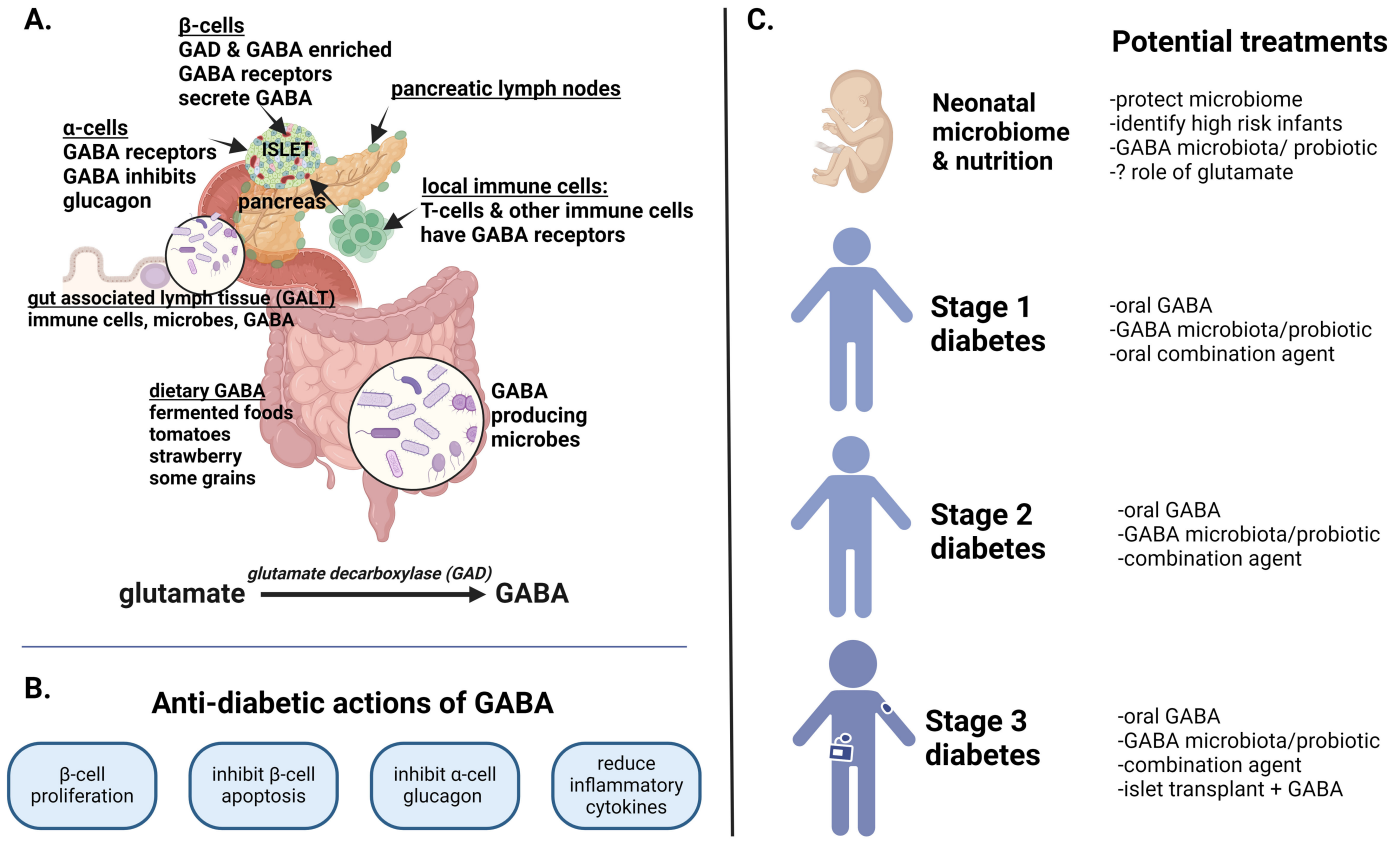
GABA in health and diabetes. This figure summarizes the role of GABA in the entero-pancreatic system, its anti-diabetic actions and potential as a therapeutic agent in type 1 diabetes (T1D). **(A)** GABA is synthesized from glutamate by glutamate decarboxylase (GAD65) which is also a T1D autoantigen. GABA is uniquely concentrated in β-cells but is also consumed in foods and produced by select GAD-containing microbiota in the upper and lower intestinal tract. From birth, gut associated lymph tissue (GALT) within the lamina propria are intricately involved in bodily defenses against autoimmunity and inflammation. **(B)** The key anti-diabetic actions of GABA are presented as validated in numerous preclinical rodent and human islet studies. In children with new onset T1D, oral GABA, with or without recombinant GAD, reduced serum glucagon as well as inflammatory cytokines. **(C)** The potential therapeutic role of GABA in T1D is shown from birth through stage 3 diabetes. The perinatal acquired microbiome is pivotal to lifetime immune defense. Whether GABA producing microbiota or glutamate have salutary immune actions is unexamined. In stage 1 diabetes (asymptomatic autoimmunity), GABA supplementation or precision probiotics might restrain the autoimmune process, particularly if GABA/GABA-producing microbiota are deficient. Combination therapy with a low risk oral therapy such as an islet antigen or anti-apoptosis agent might further preserve or expand β−cell mass. In stage 2 diabetes (autoimmunity with dysglycemia), GABA supplementation, with or without more potent combination therapies, might hamper autoimmune destruction. Possible co-therapies, noted to be effective in diabetic rodent studies, include oral T1D antigens, GLP-1 agonists, and positive allosteric modifiers that augment GABA action. Longer acting GABA formulations could also improve efficacy. In stage 3 diabetes (insulin dependent), higher dose GABA along with combination agents that preserve β-cell mass or induce β-cell proliferation are a consideration (anti-apoptotic agent, low dose immune therapies, GLP1 agonists, GABA receptor agonists). Finally, in humanized rodent diabetic models, GABA preserves implanted human islets while promoting β-cell proliferation - whether this has application for human islet transplant survival is intriguing.

Outside of the CNS, GABA is highly concentrated in the pancreatic islet wherein it has autocrine and paracrine actions to regulate β-cell insulin secretion and inhibit α-cell glucagon release. Well-known communal microbiota also produce GABA (18, 19). Rodent models have demonstrated reversal or prevention of diabetes with oral and intraperitoneal GABA treatments (20). Combination therapies of GABA with β-cell antigens, antiapoptotic agents, and immunotherapies show additive actions (21–23). In diabetic NOD-scid-γ (NSG) mice, GABA promotes β-cell neogenesis in human islet cell implants and reverses diabetes (24). In children with new onset T1D, low-dose, twice daily oral GABA, with/or without GAD-alum antigen stimulation, inhibited glucagon and reduced Th1 inflammatory cytokine release. Taken together, these studies support a unique role for GABA as a naturally derived oral agent with multifarious anti-diabetic actions. Given its excellent safety and tolerability, higher GABA doses, longer-acting preparations or combination therapies may bear salutary actions in stage 1, 2 or 3 diabetes (**Figure 1**). In this review, the potential role of GABA as an endogenous and exogenous disease modifier in T1D is presented.

## 2 Pancreatic GABA in diabetes

### 2.1 GABA in the pancreatic islet

GABA is present in assorted peripheral (non-CNS) tissues including pancreas, gonads, placenta, uterus, gastrointestinal tract, lymphatic and adrenal medulla (25, 26). Pancreatic β-cells have distinctly inordinate concentrations of GABA that are comparable to CNS tissue content (27). Several recent reviews underscore the role of GABA in the pancreatic islet (15, 28, 29). In human β-cells, GABA is synthesized from glutamate by the pyridoxal phosphate dependent enzyme GAD65, which is also a key diabetes autoantigen. GAD67 is an isoform of GAD65 found exclusively in mouse β-cells and brain, and both isoforms are present in rat β-cells (30).

Two GABA receptors are recognized. The GABA-A receptor (GABAAR) is a heteropentamer that functions as a fast-acting chloride channel, thereby altering membrane polarization. It is the primary GABA receptor in the human islet (31). Following GABA binding, there is efflux of chloride in β-cells (hypopolarization) but the opposite flow of chloride occurs in α-cells (hyperpolarization) (13). The GABA-B receptor (GABABR) is an inhibitory two-subunit G-protein receptor that reduces cAMP and modulates Ca^+2^ channels. Under basal conditions, human β-cells express only one of the two functionally necessary GABABR subunits. Modifiers that increase β-cell cAMP, such as forskolin, induce expression of the second subunit yielding functional activity of GABABR (decreasing insulin secretion) (32). Hence, while both GABA receptors are available in the human β-cell, GABAAR are functionally predominant. Regarding GABAA receptor affinity, human β-cells retain two main pentamer subunit subtypes; the stoiochiometry and arrangement of these subunits determines pharmacological selectivity regarding potential agonists and antagonist therapies (33, 34).

Autocrine and paracrine mechanisms account for the regulatory actions of ambient GABA on β- and α-cell function (15, 28, 29). By most accounts, α-cells are devoid of GAD, although this view has been disputed (35). Whether paracrine stimulation of δ-cell somatostatin secretion by GABA inhibits β-cell insulin release is unclear (35). Initial islet studies suggested the co-release of GABA and insulin by exocytosis and that the process was mediated via GABAA receptors. At 6 mM glucose, a GABAA receptor antagonist inhibited insulin secretion (31). Using patch clamp recording and PCR analysis of human islets, the authors demonstrated the presence of GABAA receptors on β-cell, δ-cells and α-cells implicating autocrine and paracrine roles for GABA. Using dynamic hormone secretion measurements in donor islets, GABA was later shown to regulate β-cell insulin release in an oscillatory pattern that was not glucose-dependent (35). GABA accumulates in the cytosol (rather than vesicles) and is secreted via volume regulatory channels. The autocrine action of GABA on β-cell insulin release was inhibitory. GABA attained local (interstitial) concentrations of 10 μM and patterned with the known oscillatory release of insulin. These investigations point to a stabilizing role of GABA in the dynamic regulation of β-cell insulin release. Menegaz and colleagues also demonstrated that T2D and T1D donor islets were 75% and 85% depleted of GABA, respectively, despite no difference in GAD65 content (35). The T2D islets lacked pulsatile insulin release until cellular GABA levels were restored by inhibiting GABA catabolism. Using single cell transcriptomics, islet cells with multiple hormone mRNA expression have been identified in human pancreatic islets (36). These mixed identity islet-cells most often express insulin/glucagon combinations but may also include somatostatin. In diabetic islets, glucagon predominant cell types are more frequent compared to controls. As to why the islet has mix-identity cells, the investigators underscore that the plasticity of the pancreatic islets (37), numerous regulatory factors, including GABA, and patterns of cellular neogenesis or dedifferentiation are all under investigation

Rodent and human islet studies demonstrate the complex autocrine and paracrine signaling that underpin nutrient-responsive crosstalk amongst α-, β- and δ- cells. Lesser-studied components include pancreatic polypeptide-secreting gamma cells and ghrelin-expressing epsilon cells which form <1% islet content. Each islet has a capillary and neural network that provides intimate connectivity with the immune system, gut, liver and CNS (17, 38–41).

Aside from receptor-mediated regulation by GABA, the metabolism of GABA via the intracellular GABA-shunt and TCA cycle further modulates β-cell GABA content and its energy metabolism. Beta-cells metabolize cytosolic GABA via the GABA shunt to meet cellular metabolic demands as the islet responds to the fluctuating energy shifts of the fasting and fed states (42).

### 2.2 Preclinical studies: GABA in diabetes

In numerous studies using diverse diabetic rodent models, GABA prevents and/or reverses hyperglycemia. Soltani et al. reported several gainful actions of GABA on β-cell mass, immune function, and clinical diabetes in two diabetic mouse models and also in INS-1 rat insulinoma cells (20). GABA increased BrdU^+^ labelled β-cells 5-fold in CD1 mice following two i.p. injections of GABA (20 μmol/mouse over 48 hours). In the multiple dose STZ-diabetic (MDSD), daily i.p. injections of GABA(20 μmol/mouse, i.p.) for 7-days prior to STZ prevented hyperglycemia, increased serum insulin, decreased glucagon, restored β-cell mass and normalized α-cell mass. In the NOD mouse, a spontaneous immune-mediated diabetes model, treatment with GABA was preventative. GABA led to an abatement of insulinitis (lymphocyte infiltration), β-cell mass expansion and normalization of hyperglycemia (after *i.p.* glucose challenge). GABA treatment reduced MDSD- related inflammation by lowing cytokines (IL-1β, TNF-α, INF-γ and IL-10) and reducing LPS^+^IFN-γ-stimulated splenic CD4^+^ and CD8^+^ cell numbers (20). Tian, et al. demonstrated that treatment of prediabetic NOD mice with GABA (delivered by subcutaneous pellet) from 6-34 weeks of age inhibited progression to overt diabetes by 70% and decreased GAD-specific INF-γ-secreting T-cells by 39% (43). Other rodent models also corroborate a salutary response to GABA in diabetes(median dose 1500 mg/kg/day, range 0.25-4500) (20, 22, 24, 44–52).

The anti-diabetic action of GABA has been studied in combination with other agents. Combination GABA with GAD immunization increased the duration of syngenic β-cell survival in diabetic NOD mice from 1 week in control-diabetic mice to 10 weeks with maximal GABA doses (GABA 6 ml/ml in drinking water + 100 mg GAD immunization) (23). GABA with proinsulin immunization corrected hyperglycemia in newly diabetic NOD mice when compared to either agent alone (49). At the highest GABA doses (20 mg/ml in water) plus proinsulin immunization, diabetic mice achieved normoglycemia with 4/9 mice remaining normoglycemic for up to 50 weeks post onset of diabetes. Combined GABA plus proinsulin reduced insulinitis, increased β-cell replication and improved splenic Treg responses compared to monotherapy. In NOD mice prior to diabetes onset (4-6 weeks old), combination rapamycin (1 mg/kg daily) and GABA (~200 mg/kg/day divided twice daily) delayed the onset of diabetes for the entire 12 week experimental period, whereas with monotherapy 20% of the mice acquired diabetes (53). In overtly diabetic NOD mice, co-therapy with rapamycin and GABA was superior to monotherapy in reducing hyperglycemia and retaining β-cell function. In INS-1 cells and human pancreatic islets, combination therapy of GABA with a GLP-1 agonist (exendin-4) led to a reduction in cytokine-induced apoptosis and improved glucose-stimulated insulin release. Moreover, the anti-apoptotic actions of SIRT1 and α-Klotho expression were normalized with GABA plus exendin-4 (54).

Finally, in severely diabetic NOD mice, low-dose anti-CD3 (35 mcg) and lesgaberan, a GABA-B receptor agonist (0.08mg/ml in drinking water), rapidly lowered blood glucoses and preserved functional β-cells over a 25-week treatment period (21). After discontinuing treatment, mice were monitored for an additional 25 weeks. The co-therapy group was 83% relapse-free compared to 30% in the anti-CD3 monotherapy group. In a separate report, Tian et al. found that treatment of diabetic NOD mice for 25 weeks with low dose anti-CD3 treatment plus a GABA-A receptor agonist (homotaurine) reversed hyperglycemia and improved the percent of relapse free mice post treatment: 60% with combined therapy, 30% with anti-CD3 monotherapy and 10% with homotaurine alone (55).

Notably, GABA has shown anti-diabetic actions in diverse T1D rodent models, including NOD mice, multiple low dose STZ mice as well as humanized rodent models such as the NOD/Lt-SCID-IL2rg or NSG mouse (55–57). Concerning the NOD mouse, GABA not only forfends against diabetes onset but also reverses overt diabetes (20). As discussed in section 4 regarding GABA dosing and safety, to date, experimental rodent doses of GABA are comparatively higher and of longer duration than oral human dosing. Furthermore, conflicting or negative GABA effects were apparent when lower GABA doses were used in mice (44, 58). As concerns treatment of T!D, longer acting preparations of GABA, co-therapy with GABA receptor agonists, positive allosteric modifiers (59) or complimentary antidiabetic agents (discussed above) could potentially overcome a need for higher GABA doses to achieve efficacy.

### 2.3 GABA promotes β-cell proliferation and survival

Loss of β-cell mass due to a reduction in β-cell proliferation/regeneration and accelerated β-cell apoptosis are synergistic processes leading to the clinical manifestations of TID. Therapies that invigorate β-cell replication and reduce β-cell destruction may favorably improve the diabetogenic imbalance of cell growth/cell demise. These therapies are relevant to the survival of islet cell transplants as well as *in situ* β-cell function. GABA promotes β-cell growth and survival (24, 60–62). Mechanistically, via an autocrine route, GABA-mediated membrane depolarization (via GABAAR) in β-cells stimulates calcium influx via voltage-gated, calcium channels. The subsequent activation of the growth promoting Ca^2+^ dependent P13K/Akt pathways in INS-1 cells and isolated rodent and grafted human islets leads to increased β-cell proliferation and survival (20, 24). This GABAAR mediated process is potentiated by augmented expression of β3 receptor subunits as shown in the partial pancreatectomized mouse diabetes model (63). Humanized rodent models have advanced our understanding regarding the remarkable proliferative potential of human islets (57, 64).

Elevated TxNIP increases oxidative stress in β-cells and other tissues via thioredoxin (65, 66). In mouse islets from STZ-diabetic mice treated for 13 weeks with GABA (6 mg/ml in drinking water), the anti-apoptotic action of GABA was linked to TxNIP (67). They reported that both GABA and GLP-1 reduced hyperglycemia-associated increases in TxNIP through a common pathway (cAMP-β-cat) . If the effect of these agents is additive, then the combination of GLP-1 and GABA in T1D warrants investigation. Others have identified the role of SIRT-1 and α-Klotho in mediating the anti-apoptotic actions of GABA in the β-cells (47, 54).

Given the abundance of islet non-endocrine cells with pancreatic lineage such as exocrine cells from acinar or epithelial duct, the neogenesis of these cells into insulin-producing β-cells presents an enticing treatment for T1D. However, low-dose GABA over months failed to induce neogenesis of β-cells from ductal tissue based on lineage-labelled ductal tissue in Sox9CreER;R26R^yfp^ mice (68). Another experimental approach to β-cell insulin deficiency would be to induce transdifferentiation of α-cells to functional β-cells with GABA. This was accomplished by Ben-Othman, et al. (44). Experiments with wild-type mice showed a dose-dependent increase of insulin^+^ β-cell mass with 1-5 mg/kg GABA that persisted at a much lower dose of GABA (250μg/kg). In a related study, when C57BL/6J mice, rendered diabetic by STZ, were treated for 8 weeks with GABA (250μg/kg), blood glucose concentrations declined in concert with a ~3-fold increase in plasma insulin, yet plasma glucagon was unaltered. By histological staining, neither pancreatic β-cell nor α-cell mass was altered by GABA treatment alone. A nearly two-fold increase in α-to-β cell conversion was observed. The results of these studies could not be replicated (69). The discordant conclusions between labs could be consequent to heterogeneous experimental protocols to measure α-cell and β-cell transdifferentiation, and other such variables as mouse strains, diets, gut microbiota, and duration of GABA treatment. Worth considering, the three research groups aforementioned used GABA doses that were logarithmically lower than most *in vivo* GABA protocols.

In an attempt to resolve different experimental conclusions regarding GABA and β-cell regeneration, especially α- to β- transdifferentiation, von Herrath et al. independently conducted a series of experiments using similar GABA doses, additional delivery methods as well as assiduously reproducing identical experimental conditions (70). They were unable to demonstrate α- to β- transdifferentiation by GABA, as well as, no effect on glucose homeostasis or α-cell/β-cell mass in normal or diabetic mice. However, there is an apparent dose-dependent trend that GABA decreased α-cell mass and the α-to β- ratio in the wild type mice.

### 2.4 GABA inhibits glucagon

Glucose control in diabetes is regulated in part by glucagon, not only through paracrine intra islet cell communication, but also through peripheral effects on hepatic, adipose and neural metabolism (17, 71, 72). Hyperglycemia triggers β-cell insulin release and suppression of α-cell glucagon secretion. Using rodent islets, Xu, et al. found that insulin secretion induces an Akt kinase dependent translocation of GABAA receptors to the membrane of pancreatic α-cells that augments the response to paracrine release of GABA from β-cells. The result is GABA-mediated membrane hyperpolarization and subsequent inhibition of glucagon secretion (73). GABA-deficient islets did not exhibit appropriate glucagon inhibition in response to increasing glucose concentrations *in vitro* (74), inferring that GABA is directly involved in the suppression of glucagon secretion in α-cells. Based on immunofluorescence studies in STZ-treated mice, daily intraperitoneal GABA (10 μg/kg) for 12 days thwarted the 7-fold rise in α-cell mass which transpired in the control-diabetic group and also preserved β-cell mass (75). The α-cell mass expansion in STZ mice likely develops in human T1D; for example, following the onset of T1D in humans, there is a progressive increase in serum glucagon for at least one year and sometimes 3-5 years thereafter (76–79). In diabetic animals, the effect of exogenous GABA on circulating glucagon and/or α-cell mass are conflicting. There was an approximate threefold reduction in serum glucagon in several studies (20, 75), but no change was noted by others (80, 81). As for the latter two studies, one involved rats and the other used a very low GABA dose (0.25mg/kg) - these experimental disparities could account for the conflicting findings. An excess of glucagon relative to insulin characterizes the metabolic dysregulation and hyperglycemia of diabetes. Treatment of children with T1D with low dose, twice-daily oral GABA, with and without GAD-alum, for 12 months reduced circulating glucagon without preserving serum c-peptide (82). In this trial, a secondary finding buttresses a role for glucagon in glycemic control: there was a significant relationship between fasting glucose and fasting glucagon. Moreover, at 12 months, there was an even more robust association between area under the curve (AUC) glucose and AUC glucagon following a mixed-meal challenge. Both of these glucagon-glucose relationships do not establish causation, yet provide intimations that compel further study.

### 2.5 GABA is anti-inflammatory

Type 1 diabetes is characterized by a multipronged inflammatory assault notable for infiltration of the pancreatic islet with autoreactive CD4^+^ and CD8^+^ T cells and macrophages begetting insulitis and β-cell demise. Antibodies to GAD65 and other β-cell antigens are present years prior to dysglycemia and overt symptomatic diabetes (4). Peripheral blood mononuclear cells (PBMC) release pro-inflammatory cytokines and chemokines that accelerate the process. Identifying safe immunomodulatory interventions to slow/abort the T1D autoimmune process or protect transplanted islets from immune destruction is imperative (14).

GABAARs are expressed in various immune cells, including T-cells and peripheral blood mononuclear cells, and are known to exert immune-inhibitory actions (43, 83, 84). Human T cells, dendritic cells, NK killer cells, and monocytes, also contain the enzymatic components for GABA production (including GAD) and catabolism (GABA transaminase) (85, 86). In NOD/*scid* mice, daily GABA (600 μg/day by subcutaneous pellet) inhibited the adoptive transfer of T1D indicating suppression of effector-T cells. In addition, continuous low-dose GABA for 30 weeks reduced the onset of diabetes in NOD mice: 90% of control mice developed diabetes compared to the 20% of those treated with GABA (43).

GABA suppresses the formation of IL-12 by macrophages, and IFN-γ by CD8 T-cells, underscoring its anti-inflammatory role of reducing cytokine production (20, 87). In rat INS-1 cells versus human β-cells, GABA attenuates cytokine-induced (IL-1β, TNF-α, INF-γ) apoptosis 75% and 30%, respectively; these actions were potentiated by a glucagon-like peptide-1 (GLP-1) (54). As recently reported, ambient glucose or insulin modulate the effect of GABA on inflammatory cytokine release in human CD4+T-cells (88). In children with new onset T1D, oral GABA, with or without GAD65-alum, curtailed the Th1 proinflammatory response relative to placebo (89). Following antigen stimulation of PBMC with GAD65, GABA/GAD treatment showed a blunted (absent) rise in INFγ and TNFα compared to the statistical increase in both proinflammatory cytokines in a placebo group from 0-12 months (p<.05).

## 3 GABA and the microbiome

### 3.1 GABA producing microbes

The intricate entero-pancreatic biology of GABA, ingested or synthesized by microbial glutamate decarboxylase(GAD), is conceivably germane to T1D pathophysiology. As aforementioned, GAD65 is concentrated in the β-cell (15) and found in discrete enteric bacteria (19). In microbiota, an intact GAD operon (includes both gadB or gadA plus the glutamate/GABA antiporter) is requisite for GABA metabolism (90) and acid/base tolerance (91). In the gastrointestinal tract, lactic acid bacteria such as L. brevis and L. reuteri (phyla Firmacutes), as well as bifidobacteria (phyla Actinobacteria) including B. adolescentis and B. dentium, are acclaimed GABA producers (92–94). Of 135 strains of Lactobacillus and Bifidobacterium from human donor enteric/salivary/vaginal specimens, 58 srains produced GABA from glutamate *in vitro* (94). The authors confirmed the presence of gadB/gadC genes in the bacteria and noted *in vitro* GABA production rates of 50-6000 mg/L in timed incubations. Standwitz, et al. identified a previously unculturable gram positive bacterium (KLE1738) that required a common GABA-producing gut microbe -Bacteroides fragilis- to grow *in vitro* (19). Genome-based metabolic modelling uncovered genera of enteric bacteria capable of consuming or producing GABA. This work highlights the overlapping roles of GABA in microbiota as an energy source or pH modifier via the GABA shunt (95) versus its role in neuroendocrine signaling and immune regulation (96). The question whether GABA forming microbiota can alter plasma GABA is unresolved: two germ-free models employing metabolomics support this premise (97, 98) whereas another germ-free rodent study did not (99).

### 3.2 Microbial GABA in diabetes - preclinical studies

Several studies have examined the effect of GABA-producing microbes in streptozocin (STZ) diabetes. A single-dose streptozotocin (STZ) model was employed which causes abrupt chemically mediated β-cell destruction (100) and, hence, the results to not entirely translate to immune-mediated diabetes. Marques et al., treated STZ-diabetic rats with *Lactobacillus* GABBDPC6108 or GABA alone (2mg/kg/day or versus 200 mg/kg/day in drinking water) over 9 weeks (81). The investigators confirmed that the microbe-treated rats retained live, GABA-producing *L.brevis* in fecal samples at study end. Concerning diabetic parameters, there was a 26% decrease in blood glucose in the diabetic *L. brevis*-treated rats. GABA-treatment was associated with a 12-15% decrease in blood glucose. The serum GABA level was unchanged in the low-dose GABA group but increased 34% in the high dose GABA group. The investigators concluded that the nominal reduction in glucose by *L. brevis* or oral GABA was likely due to the massive β-cell destruction in their non-inflammatory STZ-dose rat model. Insofar as the effects of microbial-produced GABA is anti-inflammatory, perhaps a multiple dose STZ (MDSD) or autoimmune model, in which there is both inflammation and residual β-cells, would have revealed anti-diabetic actions in these experiments.

Using *specific pathogen-free* male C57BL/6 mice, Abdelazez et al. treated two groups of STZ-diabetic mice with different strains of *Lactobacillus brevis* (KLDS 1.0727 and KLDS 1.0373) and compared diabetes-related parameters relative to control mice and STZ-treated diabetic mice (no probiotic treatment) after 4 weeks. There was a 40% decrease in blood glucose in the *L. brevis*-treated STZ-mice compared to untreated STZ-controls (serum glucose 7mM versus 4 mM, respectively). The *L.brevis* strains were shown to contain a GAD gene and produce GABA. Proof of sustained enteric colonization with the Lactobacillus was not documented (101). In high fat-fed, insulin-resistant mice, *L. brevis* readily colonized the animals, increased insulin sensitivity, and, following an overnight fast, increased the GABA concentration in the small intestine 2.25-fold (102).

In aggregate, these STZ-diabetic rodent models showed modest metabolic actions on glucose and insulin with *L. brevis* treatment without reversal of diabetes. This supports that the primary salutary actions of microbial-GABA in T1D may be immunologic. Hence, long-term enhancement of GABA-producing microbiota, particularly in the entero-pancreatic region, may be requisite to mitigate autoimmune β-cell destruction. And, concerning the role of GUT health, many other factors, including nutrition, prebiotics, additional microbe-derived metabolites such as short chain fatty acids (SCFA), along with avoidance of unnecessary antibiotics, warrant study in T1D (16).

### 3.3 Crosstalk between gut microbiota and the pancreatic islet

The human gastrointestinal tract from the oral cavity to colon harbors distinct microbial ecosystems. Accordingly, microbes contained in a distal stool specimen, while experimentally convenient, differ considerably from proximal segments (103–105). In 21 healthy individuals age 59 ± 12.3 years who had endoscopy to obtain mucosal biopsies of the upper and lower GI tract (103) fecal microbiota by 16S ribosomal profiling did not mirror those in the upper intestinal mucosal microbiota. Noteworthy, lactobacilli (phylum Frimicutes), which includes many GABA-producing microbiota, were exclusive to the upper GI tract compared to fecal samples. Fecal GABA levels, however, correlate with Bifidobacterium abundance (phylum Actinobacteria) in healthy controls (106). In a catheterized rat model, serum GABA was measured in the venous effluent from small versus large intestine after selective ligation of abdominal arteries and veins. A two-fold increase in portal GABA concentration was found between the fasting and fed states, as well as a 50% diminishment in serum GABA in large versus small bowel effluent (99).

Concerning entero-pancreatic signaling, or crosstalk, between microbiota and the pancreas, local, as opposed to systemic, GABA levels are likely more relevant to autoimmune diabetes (107). The anatomical proximity and connections between microbiota in the nutrient-rich duodenum, gut-associated lymph tissue (GALT) and pancreatic lymph nodes (PLN) form a complex network that mediates immune tolerance (39, 108). For example, in control mice, pancreatic β-cells produce calthelicidin-related antimicrobial peptide (CRAMP) in response to microbial-derived SCFA; this response mechanism is deficient in both NOD mice and multiple dose STZ diabetes (MDSD) mice that are genetically CRAMP-negative (109). Replacing CRAMP forestalls diabetes in these rodents and is associated with reduced pancreatic immune cell infiltrates (B-cell, T-cell, and dendritic cells). This novel rodent study demonstrates that crosstalk between β-cells and the metabolites of intestinal microbiota may contribute to the immune backdrop that forfends against autoimmune diabetes. Studies in germ-free NOD,MyD88-deficientKO mice also highlight a protective interaction of commensal microbes with the immune system that reduces the incidence of diabetes (110). It is, therefore, reasonable to posit that within this enteric micro-environment, a healthy complement of GABA-producing microbes might favorably modulate T-cell immunity and islet cell function (81, 111, 112).

### 3.4 Microbial GABA and type 1 diabetes

Type 1 diabetes is associated with alterations in the composition of gastrointestinal microbiota (dysbiosis) and breakdown of the gut barrier integrity (113–115). Longitudinal analysis from the TEDDY trial of fecal microbes and their metabolites from infancy to T1D-onset has uncovered bacterial imbalances notable for reduced ratios of Firmicutes to Bacteroidetes as well as deficient enteric SCFA (116–119). Serum metabolome analysis disclosed reduced GABA levels one year before seroconversion to insulin autoantibodies (IAA), but not before the appearance of GAD antibodies (120). This observation was corroborated in the Finnish Type 1 Prediction and prevention (DIPP) study wherein elevations in glutamate (precursor to GABA) were apparent prior to seroconversion. And, in a salient case study, an 8-fold spike in serum GABA and 13-fold increase in glutamate preceded the appearance of GAD antibodies by 2.5 years (121). The significance of these GABA/glutamate trends are unknown but may reflect compensatory immunomodulation, diet, microbiota, infection or unknown exogenous factors. Microbial dysbiosis has been implicated as a key element, in concert with genetic predisposition and environmental factors, which underpin the pathoetiology of T1D. It follows that a deficiency of GABA-producing microbiota, particularly in the duodenum, may be a component of diabetic dysbiosis. GABA receptors are abundant in the intestinal tract and on T-cells where anti-inflammatory actions are recognized (94, 113). T cells express functional GABAA receptors that are responsive to low dose GABA (43). As follows, GABA production by the microflora in the metabolically active small intestine could conceivably lessen pathogenic autoreactive T-cell responses in gut-associated lymphoid tissue (GALT) and/or pancreatic lymph nodes (117, 122).

A straightforward approach to dysbiosis in T1D is the introduction of probiotics (123, 124). Most human trials have tested the benefits of combinations of Lactobacillus (phyla Firmacutes) a bifidobacteria (phyla Actinobacteria) (124, 125). While GABA production was not the focus of these investigations, many lactobillus and bifidobacteria express GAD and, thereby, produce GABA (19, 90, 92, 94, 126, 127).

### 3.5 Microbial glutamate decarboxylase (GAD) and autoimmunity

GAD65 is a pyridoxal (B6)-dependent decarboxylase. The enzyme can alternate between an antigenic apoGAD65 format (no attached B6) versus its active and less antigenic holoGAD65 format (B6 bound). This contrasts with the non-antigenic holo- GAD67 that is only B6- bound (128). The hypothesis that GAD-containing microbiota might trigger an autoimmune attack against β-cell GAD65 was considered based on similarities in human versus bacterial GAD epitopes in the B6 binding region of GAD (129). The antigenic, pyridoxal linkage site of GAD65 in GABA-producing gut microbes aligns closely with human GAD65 such that microbial GAD could conceivably sensitize enteric T-cells to GAD65 leading to the pathogenic immune destruction of β-cells. In this model, B6 deficiency might enhance exposure of the antigenic catalytic site of GAD to autoimmune detection (130). Nevertheless, increased vitamin B6 intake was not protective against T1D progression in the TEDDY study (131).

### 3.6 Glutamate

Glutamate, the enzymatic precursor for GABA, constitutes about 10% of dietary amino acid content. Analogous to GABA, glutamate is a CNS neurotransmitter with additional actions outside the CNS. Glutamate receptors are widespread, found particularly in pancreas, adrenal gland, developing cartilage, gastrointestinal tract and lymphocytes (132). For unknown reasons, free glutamate is uniquely concentrated in human milk (133), several-fold higher than other amino acids . The free glutamate intake of breast-fed infants is 36 mg/kg compared to 0.7 mg/kg from dairy milk formulas; protein hydrolysate preparations provide 170 mg/kg. Enteral glutamate is rapidly oxidized for intestinal metabolic energy in piglets (134), preterm infants (as measured with stable isotopes) (135) and adults. Glutamate is, furthermore, the widely applied, unami food enhancer (mono-sodium glutamate or *MSG*). Inasmuch as oral glutamate is metabolized rapidly by enterocytes, there was no measurable rise in systemic GABA levels following an oral dose of glutamate (136, 137). Concerning glutamate metabolism and signaling in pancreatic islets, extracellular uptake of glutamate by AMPA receptors augments *a*-cell glucagon release (138). In β-cells, glutamate potentiates glucose and incretin-stimulated insulin signaling and islet survival via NMDA receptors (139). The intracellular metabolism of glutamate in β-cells involves mitochondrial glutamate dehydrogenase, glutamate decarboxylase (GAD), glutamine synthetase, and the synthesis of glutathione (140). The glutamate NMDA receptor is a proposed drug target for diabetes (140–142).

## 4 GABA dosing and safety

**Figure 2:**
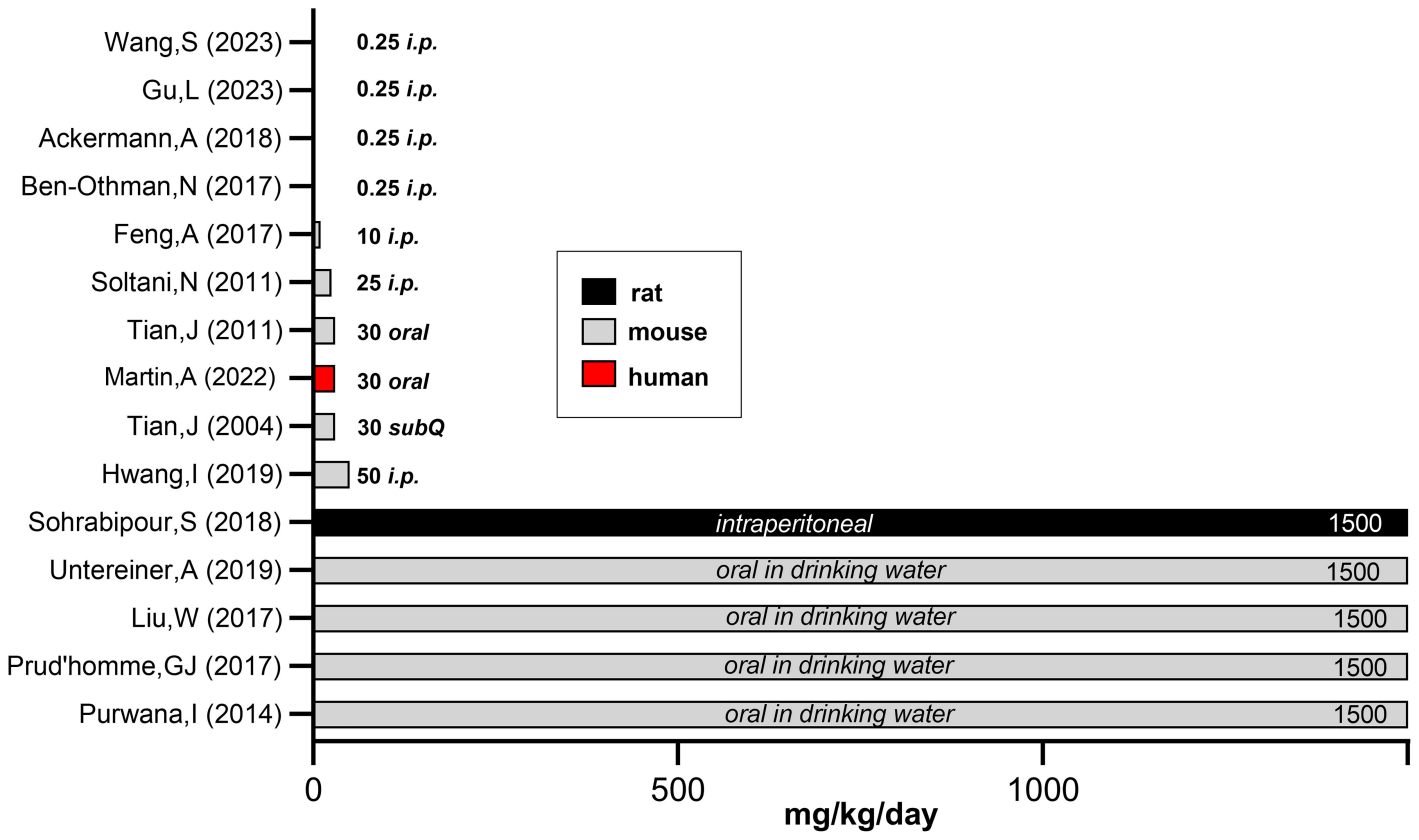
Comparison of experimental GABA doses used in rodent versus human studies. To compare the experimental GABA doses (mg/kg/day) used in rodent versus human studies, we estimated daily water intake and tabulated average adult rodent weights. When GABA was added to drinking water or given by injection, the daily intake approximated 1500 mg/kg/day based on estimated daily water consumption (148). This calculation does not take into account that diabetic animals have polydipsia, thus the actual GABA dose is vastly underestimated. Mouse body weights - unless noted by investigators in the methods section- were based on species and the average, non-diabetic weight in healthy animals. **Figure 2** references (Y-axis): Wang, et al. (68), Gu, et al. (80), Ackermann et al. (69), Ben-Othman et al. (44), Feng et al. (75), Soltani et al. (20). Tian et al. (23), Martin et al. (82), Tian et al. (43), Hwang et al. (45), Sohrabipour et al. (48), Untereiner et al. (51), Liu et al. (22), Prud’homme et al. (47), Purwana et al. (24). Figure adapted from "A randomized trial of oral gamma aminobutyric acid (GABA) or the combination of GABA with glutamic acid decarboxylase (GAD) on pancreatic islet endocrine function in children with newly diagnosed type 1 diabetes," by Martin A, Mick GJ, Choat HM, Lunsford AA, Tse HM, McGwin GG Jr, and McCormick KL. Nat Commun. 2022 Dec 24;13(1):7928, Supplementary Data, Figure 6 (https://doi.org/10.1371/journal.pone.0197160).

GABA is a water soluble, non-protein amino acid (C4H9NO2). It is considered a dietary supplement in the USA (143) and a pharmaceutical in Europe (144). The Dietary Supplement Label Database (https://dsld.od.nih.gov) records over 1500 GABA-containing supplements with daily doses ranging from 45 mg to 3000 mg/day, and most at 500-750mg/day (143). A toxicity study in rats administered oral GABA (500-2500 mg/kg/day) for 13 weeks and found no significant abnormalities in behavior, weight gain, or blood indices including general chemistries, glucose, renal function, hematology and liver function. Postmortem organ histopathology and weights were normal (145). The highest reported oral dose of GABA involved 14 adults (8 gram/kg/day divided into 4 doses) for up to 2 years and was well-tolerated (146). In healthy adults, single GABA doses of 5 gm, 10 gm or 18 gram/day for 4 days was without serious adverse side effects (147). **Figure 2** presents a comparison of experimental daily GABA doses (mg/kg) in rodents and one human clinical study. Of importance, oral administered GABA does not cross the blood brain barrier (143), although this viewpoint may need further analysis in neonates (149).

Using immunoassay, adults with T1D had plasma GABA levels of 649 ± 42 nM compared to 501 ± 32 nM in controls (87). In T2DM, plasma GABA concentrations were 480 ± 28 nM in T2D compared to 516 ± 30nM in non-diabetic controls (33). In a clinical trial, baseline, fasting GABA levels were 248 ± 86 nM by LC-MS/MS in children with T1D (82). Peripheral blood GABA levels, as measured by HPLC, do not vary significantly by gender or exercise (150). Using LC-MS/MS, fasting GABA was 10 ng/ml (97 nM) in 12 healthy volunteers (151). Following a 2 gram oral dose of GABA, there was a rapid rise in plasma GABA (t_max_: 0.5-1 hour, C _max_ 6.7 μM, t_1/2_ = 5 hours). With repeated dosing of 2 grams GABA three times per day for 7 days (~85 mg/kg/day), GABA levels were at steady state. For comparison, in mice, GABA treatment (6 mg/ml in drinking water for ten weeks=1500mg/kg/day) raised plasma GABA five-fold over a baseline of 47.4± 4.8 ng/ml (460 nM) (51). In another report, fasting GABA levels were 16 ng/ml (155nM) in 11 male adult volunteers when measured by LC/MS/MS. Following ingestion of 888mg GABA in 1 liter of water, the pharmacokinetic variables were: t_max_ (h) = 0.5 and the C_max_ (ng/ml) = 75. Interestingly, ingestion of pureed tomatoes (innately high in GABA) that contain a comparable 888 mg dose of endogenous GABA, the GABA kinetics were: t_max_ (h) = 0.36 and the C_max_ (ng/ml) = 184.

In all pharmacologic interventions, a threshold concentration must be attained for efficacy. Hence, thrice-daily oral GABA, which is a practical outpatient regimen, and/or higher doses, is suggested given the short half-life of GABA. As emphasized by Kaufman’s lab concerning the clinical utility of oral GABA, there is evidence that the GABAA receptor EC_50_ is of low affinity (50-400 μM) (55). By patch clamp technique, human islets attained maximum channel opening at 100-1000 nM GABA with desensitization occurring above this concentration range (33). The interstitial GABA concentration in the islet is unknown, yet reason dictates that continuous exposure or frequent and higher dose GABA may be required for efficacy. Alternative therapeutic options include longer acting receptor agonists such as lesogaberan, a GABA-B receptor agonist (21, 152), or homotaurine, a GABA-A receptor agonist (55). Other long- acting GABA formulations are in clinical trials (144) or early development (153, 154). Tian et al. demonstrated the role of positive allosteric modulators such as alprazolam to augment and/or prolong GABA actions (59).

## 5 Clinical studies using GABA in diabetes

### 5.1 GABA and GABA/GAD65-alum clinical trial in children with recent onset T1D

The GABA and GABA/GAD65 trial (82) was the first human, prospective, double blind, placebo-controlled and randomized clinical trial of oral GABA (with and without GAD65-alum) in new onset type 1 childhood diabetes. The investigators hypothesized that treatment with oral GABA, or a combination of GABA/GAD65-alum, would halt or slow the progression of new onset type 1 diabetes (T1DM) by some/all of the following mechanisms: 1) increasing endogenous insulin secretion, 2) suppression of glucagon release, 3) dampening the T-cell mediated autoimmune process. This single center, one-year trial enrolled 97 children with T1D within 6 weeks of diagnosis (NCT02002130). Interventions included oral GABA (1 gram/M2/day up to a maximum of 1.5 gram/day or approximately 30 mg/kg/day, see **Figure 2**) divided into two daily doses with or without two GAD-alum injections (20 mcg/dose)-one at baseline and the other at one month. The FDA constrained the permissible

GABA treatment dose given that this was the first human trial, no less in children. While the primary outcome (preservation of fasting/meal-stimulated c-peptide) was not attained, the secondary outcome (reduction of glucagon) was demonstrated in the GABA/GAD group. Importantly, the safety and tolerability of oral GABA in children was confirmed. Considering the low oral GABA dose administered, it was not unforeseen that only glucagon inhibition was detected, corroborating the paracrine inhibitory effect of β-cell GABA on α-cells. Overnight fasting plasma GABA levels did not differ between T1D and controls in this pediatric trial, not unexpected with the short half-life of GABA. In contrast, adults with T1D had 13% higher fasting blood GABA levels compared to controls (87). Strengths of this T1D trial were the recruitment of young patients within 5 weeks of diagnosis and the inclusion of a combination antigen (GAD-alum) plus GABA study group (23). Limitations of this study were the low-dose of GABA and twice daily dosing to encourage study drug adherence. Compliance was measured by pill counts of returned study drug. The average compliance was 83% with 20% of patient visits recording <50% compliance over the study course. Based on a half-life of 5 hours after a two gram oral GABA dose (151), in combination with non-ideal study drug adherence, it is possible that islet GABA exposure was insufficient to achieve an anti-diabetic effect. Future human GABA trials could entail longer-acting preparations, higher doses, GABA agonists or precision GABA-producing probiotics. As previously discussed, preclinical studies support combination therapies (23, 54, 67, 155).

### 5.2 GABA and GABA/GAD65-alum alters Th-1 cytokine response in children with recent onset T1D

In the same cohort as the GABA/GAD-alum study (82), the potential immune effects of GABA treatment, with or without GAD65 immunization, were examined (89). B ased on cytokine responses in peripheral blood mononuclear cells following polyclonal and GAD65 antigen re-challenge, proinflammatory Th1 cytokine responses were attenuated in both the GABA and GABA/GAD65-alum groups over 12 months.

Peripheral blood mononuclear cell (PBMC) mRNA expression was measured following polyclonal stimulation with anti-CD3/CD28 Dynabeads. GABA treatment decreased IFNγ expression at 5 months compared to placebo and with GABA/GAD at 12 months (p<0.05). At 12 months, GABA increased expression of FOXP3, a transcriptional regulator of Treg differentiation (p<.05). Using an antigen-specific recall assay to GAD65, IFNγ mRNA decreased with GABA/GAD compared to GABA alone at 12 months (p<.05).

The cytokine/chemokine response of PBMC’s was measured following antigen stimulation with GAD65 using a Milliplex MAP human cytokine/chemokine bead panel. GABA/GAD treatment showed a blunted (absent) rise in INFγ and TNFα compared to the statistical increase in both cytokines in the placebo group from 0-12 months (p<.05). GABA decreased the Th1 inflammatory chemokine CXCL10 response between 0 to 5 months but this diminishment reversed by 12 months. The placebo group, by contrast, had an increase in CXCL10 between 5-12 months (p<0.05) and 0-12 months (p<0.01).

In aggregate, by qPCR and cytokine/chemokine analysis, GABA and GABA/GAD reduced some but not all proinflammatory cytokines and chemokines consistent with an attenuated progression of the inflammatory phenotype. Subjects were next divided by high-risk haplotypes as either HLA-DR3-DQ2 and HLA-DR4-DQ8/other. The DR4 group manifested a Th1-skewed proinflammatory response in comparison to the DR3 group and responded differently to GABA alone versus GABA/GAD65-alum. Expression of IFNγ mRNA over 12 months was lower in the GABA/GAD group compared to placebo (p<0.001) or GABA alone (p<0.01) as well as compared to the same treatments in the HLA-DR4 cohort. At 12 month, GABA/GAD treatment led to decreased CXCL10 in the DR3 group compared to placebo (p<0.05) and the HLA-DR4/other GABA group. IL-2, which promotes expansion and maturation of naïve T-cell to T-eff, showed no differences with the placebo versus treatment groups.

These immune studies in PBMC from study subjects confirm the HLA-delineated immunomodulary actions of GABA and GABA/GAD65-alum in children with recent onset T1D. The results corroborate, in part, preclinical studies in MDSD mice showing that GABA decreased levels of circulating and CD4-released IFNγ, IL1β, TNFα, and IL-12 mice (20). The immunomodulary effect of GABA in NOD mice (600 mcg daily by subcut. pellet for 60 day) is also instructive (43). For example, in GAD-stimulated splenic T-cells from the NOD mice, GABA reduced INFγ formation 55%. The GABA dose used in the NOD mice (~30 mg/kg/day) (see **Figure 2**) is comparable to this clinical trial (82, 89).

Limitations of this study were the low dose of GABA and challenges with medication adherence as discussed previously. Concerning immunophenotyping of isolated PBMCs, it is evident that results do not perfectly mimic the localized immune response within the pancreatic islet. Corroborating the results in animal models of T1D treated with GABA and GAD65-alum could clarify whether the peripheral immune responses resemble the islet microenvironment.

In addition, both GABA alson and GABA with GAD65-alum treatment inhibited Th1 responses compared to placebo but showed no significant differences between the treatment cohorts. It is possible that multiple autoantigens are necessary to induce robust T cell proliferation as was shown in an analogous T1D study that used antigen recall assays and HLA delineation (5).

### 5.3 GABA trial in adults with prediabetes

In overweight adults with prediabetes, De Bie and colleagues examined the effect of oral GABA on glycemic control using a double-blind, randomized and placebo-controlled study design (NCT04303468) (156). In this well-designed trial, 52 subjects, ages 50-70 years, were given 500 mg GABA orally thrice daily versus placebo for 95 days. Prediabetes was defined by abnormal oral glucose tolerance testing (OGTT). The primary outcome was the effect of GABA on OGTT, and the secondary exploratory outcomes included continuous glucose monitoring (CGM), cardiovascular indices and sleep quality. Blood sampling included glycated hemoglobin, insulin, glucagon, GABA, glutamate and lipids. Results did not establish the primary endpoint, although there was a 0.22 mmol/L decrease in fasting glucose in the GABA group after 95 days. Other secondary outcomes were not met. Given the role of excess hepatic glucose production and reduced glucose clearance in the pathophysiology of prediabetes (157) the inhibition of glucagon by GABA, in addition to β-cell replication, could favorably improve the insulin/glucagon ratio (158, 159).

### 5.4 Efficacy of combination therapy with GABA, a dipeptidyl peptidase-inhibitor and a proton pump inhibitor in adults with T1D.

This retrospective study examined the effect of GABA (500 mg orally 2-4 times/day) in combination with one of two DPP-4i (sitagliptin or saxagliptin) and a proton pump inhibitor (PPI) (omeprazole 20-40 mg/day) in 19 overweight adults (32± 13 years of age) with insulin dependent diabetes (160). The authors based this study on their preclinical report examining the effect of GABA, DPP-4i and PPI in NOD mice (161). T1D was characterized by low c-peptide (5/19 subjects) and GAD65 positivity (14/19 subjects). Patients were identified by chart review and were divided into two subgroups: early-therapy (begun within 12 months, mean 3 months, of starting insulin) and late therapy (begun more than 12 months, mean 168 months, after starting insulin). Treatment continued for 26-42 months. There were improvements in fasting blood glucose, HgA1C, IDAA1c, total daily dose of insulin, and c-peptide. Seventy percent of patients in the early- therapy subgroup no longer required insulin but none in the late-therapy group. Moreover, despite persistently low fasting c-peptide, the combination treatment led to improvements in glycemic control and reduced total daily insulin. The authors inferred that reduced glucagon secretion may have played a role. In T2D with insulitis, beta cell failure and glucagon excess would also likely benefit from this combination therapy. Preclinical studies support this possibility (22, 49, 54, 56, 67, 155).

### 5.5 GABA levels and GAD65 antibody titers in adults with T1D

Plasma GABA levels, GAD65 antibody titers, c-peptide, and serum cytokines were determined in 128 young adults: 45 healthy controls, 60 individuals with long standing T1D and 13 individuals with new onset T1D (162). Fasting morning blood was collected for analysis and GABA was measured by LC/MS/MS. Detectible serum c-peptide was found in 20% of patients with long-standing diabetes. Plasma GABA was similar in each group and correlated positively with fasting glucose and negatively with age. The authors posit that while circulating GABA levels were the same in all groups, GABA concentrations in the entero-pancreatic region and islets may be at variance. Moreover, both dietary intake and GABA- producing microbes are additional sources of GABA that may modify the islet milieu but not be reflected by circulating concentrations (19, 127, 163).

### 5.6 GABA induces a hormonal counterregulatory response in subjects with long-standing T1D

Six adult males enrolled in an open-label, 11 day study to test the safety, efficacy, pharmacokinetics and hormonal responses (including a hypoglycemic clamp) to a long-acting oral GABA preparation (Remygen, Diamyd Medical, Stockholm, Sweden) (144). Subjects were on average 25 years old and had long-standing diabetes. In 5 subjects the baseline c-peptide was <0.01 nmol/L. Results found that the long acting GABA preparation restored the counter-regulatory response (glucagon, cortisol, and adrenaline) to hypoglycemia (clamped at 2.5 mmol/L). The authors suggest a potential therapeutic action of their GABA preparation on hormonal counter-regulation during hypoglycemia. Note that with normal to high glucose, GABA inhibits alpha-cell glucagon release (15, 164).

## 6 GABA in hybrid diabetes

T1D and T2D have overlapping features such that both have relative or veritable insulin-deficiency (with or without autoimmunity) or insulin-resistance, both of which are identified in many patients who were previously classified to one or the other binary designation. Assorted recent classification schemes have been proposed to subdivide diabetes subjects as: double-diabetes, hybrid-diabetes, type 1.5-diabetes, early onset T2D or late-onset autoimmune diabetes (LADA) (165–167). The potential efficacy of GABA in hybrid diabetes is relevant given the purported capacity of GABA to increase beta cell mass and reduce glucagon (17, 80, 168). In the high fat fed/streptozocin type 2 diabetic rat model, GABA improved insulin sensitivity and reduced expression of lipogenic genes both in diabetic rat mothers and their offspring (169). Using the same model, Sohrabipour, et al. demonstrated that GABA (1.5 gr/kg/day, IP) normalized hyperglycemia, improved insulin sensitivity (measured by insulin clamp), reduced liver glucagon receptor mRNA (but not glucagon levels), and increased muscle GLUT4 translocation to plasma membrane as well as GLUT4 mRNA expression (48). Concerning the insulin-resistant phenotype, GABA treatment also reduced diabetic rat abdominal fat compared to an insulin-treated counterpart. In an olanzapine-induced insulin resistant model, GABA treatment (50mg/kg/day, *i.p.*) decreased insulin resistance through GABA-B receptor dependent mechanisms in adipose stromal vascular tissue (170). In pancreatic donor islets from non-diabetic versus T2D individuals (171), GABA-A receptor subunits in the T2D islets were downregulated compared to controls. The authors propound that deficient islet GABA signaling/content may contribute to the hyper-glucagonemia of T2D which again reinforces a role for GABA-based therapeutics.

## 7 Conclusion

The role of GABA as a safe and inexpensive therapeutic agent for diabetes is reviewed herein. Unlike exogenous interventions, GABA is a natural compound with a distinctive physiologic role in the pancreatic islet and nutrient gut. GABA is available in select foods and over-the-counter supplements. Pharmacokinetic and safety studies demonstrate that oral GABA has a short half-life, excellent tolerability and does not cross the blood brain barrier. Insofar as GAD autoantibodies are detected early in nearly all T1D, the logical segue was that islets would be depleted of the product of this enzyme, namely, GABA. Indeed, this has been confirmed in T1D and T2D donor islets. Preclinical studies demonstrated reversal of rodent diabetes (immune or chemically-mediated) using intermittent or continuous oral GABA dosing, as well as with subdermal implants. To date, experimental animal GABA doses (mg/kg/day) are generally many-fold higher than employed in clinical studies and, in general, higher doses (**Figure 2**) were more likely to elicit a favorable metabolic response. In adults, two small cohorts ingested 20-50 times the usual over-the-counter GABA dose (~1000 mg/day) for days to months without incident. There are no long-term safety data regarding GABA treatment. New approaches are emerging to prolong the half-life and efficacy of GABA using GABA receptor agonists (21, 55), long acting formulations (144), and positive allosteric modifiers (172). These agents may obviate the need for frequent and high oral doses of GABA.

slet studies in rodent and human islets, including single-cell transcriptomics, have unveiled a multitude of paracrine and autocrine GABA actions. There is more to learn regarding the GABA’s role in modifying β-cell survival, regeneration and insulin secretory patterns. How GABA partakes in the regulatory crosstalk between the three major islet endocrine cells (β, α, and δ) is under study (15). Given its safety record and anti-inflammatory action, GABA may play a role in islet transplantation, either alone or in combination with other immunosuppressive or anti-apoptotic agents.

The endocrine and immunologic roles of GABA within the entero-pancreatic mid-gut as pertains to diet, the microbiome and the abundant gut-associated and pancreatic lymph tissue is likewise ripe for study (16, 163). A host of questions persists. Do GABA-enriched foods have health benefits? Do environmental toxins/antibiotics lead to GABA-deficient dysbiosis and reduced innate immunity? Human immune cells have GABA receptors including lymphocytes, CD4+. CD8+, PBMC, and monocytes. Do GABA-producing microbes have an immunosuppressive role concerning T1D autoimmunity? Could GABA-producing microbiota have analogous immune protective actions to SCFA-secreting microbes concerning β-cell immune protection and crosstalk in T1D? Do GABA-producing microbiota participate in primary TID prevention? To the point, disappearance of *bifidobacterium infantia* from the infant gut is implicated in the early dysbiosis of T1D (173). Of relevance, *b. infantia* is a recognized GABA-producer (111, 174). Probiotic trials frequently select Lactobacillus (phyla Firmacutes) as well as Bifidobacteria (phyla Actinobacteria) both of which contain recognized GABA-producing microbes via expressed glutamate decarboxylase (GAD). Whether microbial dysbiosis sensitizes the host immune system to GAD sequence dissimilarities between human and microbial is an alluring hypothesis (129) that deserves further consideration. Preventive management of gut dysbiosis might theoretically diminish this risk by correcting microbial imbalances and maintaining gut integrity.

GABA elicits an antidiabetic outcome by numerous routes. The fact that GABA can strikingly reverse hyperglycemia in diabetic mice, both STZ-induced and immune models, merits further clinical trials. Given the depletion of GABA in islets from patients with T1D and T2D (35), repletion of islet GABA may have pharmacologic application. Whether systemically administered GABA can replete this is unsettled, no less whether the experimentally measured islet cell deficit is indeed pathogenic. An alternative multipronged therapeutic approach would be GABA in conjunction with other immunomodulary or anti-diabetic compounds that have diverse mechanisms of action. Examples include GLP-1 agonists, DPP-4 inhibitors, TxNIP inhibitors, islet antigens, low-dose anti-CD3 antibody, and positive allosteric modifiers of GABA (13, 14, 21, 22, 59, 67).

The propitious safety profile of GABA renders early and longer-term GABA therapeutics particularly attractive, especially in stage 1 and 2 diabetes. The ongoing GPPAD-02 infant study (175) provides a paradigm for primary prevention with oral GABA. The underexplored role of endogenous GABA-producing microbiota in the immunoprotective enteropancreatic gut is apt for preclinical study and randomized controlled trials (RCT) with GABA producing probiotics in stage 1 diabetes (113). A lifetime of microbiome-protective nutritional and pharmacologic options for gut health may also defend against T1D. Combination therapy of GABA with a complimentary oral agents such as a TxNIP inhibitor or positive allosteric modifier in stage 2 T1D is an inexpensive intervention, and especially attractive insofar as the low toxicity. Based on residual β-cell function in stage 3 diabetes (176), β-cell preservation may also be feasible with longer acting or higher dose GABA formulations (82). Looking forward, GABA may have unique and previously underappreciated therapeutic benefits in TID to increase β-cell content, reduce excess glucagon and curtail the inflammatory T-cell dysfunction of type 1 diabetes.
